# Supplementary figures and images for: Serine palmitoyltransferase-mediated de novo sphingolipid biosynthesis is required for normal insulin production and glucose tolerance
Source: bioRxiv. 2025 May 17:2025.05.14.653935. Preprint. [Version 1] doi: 10.1101/2025.05.14.653935 (PMC12132366; doi:10.1101/2025.05.14.653935)

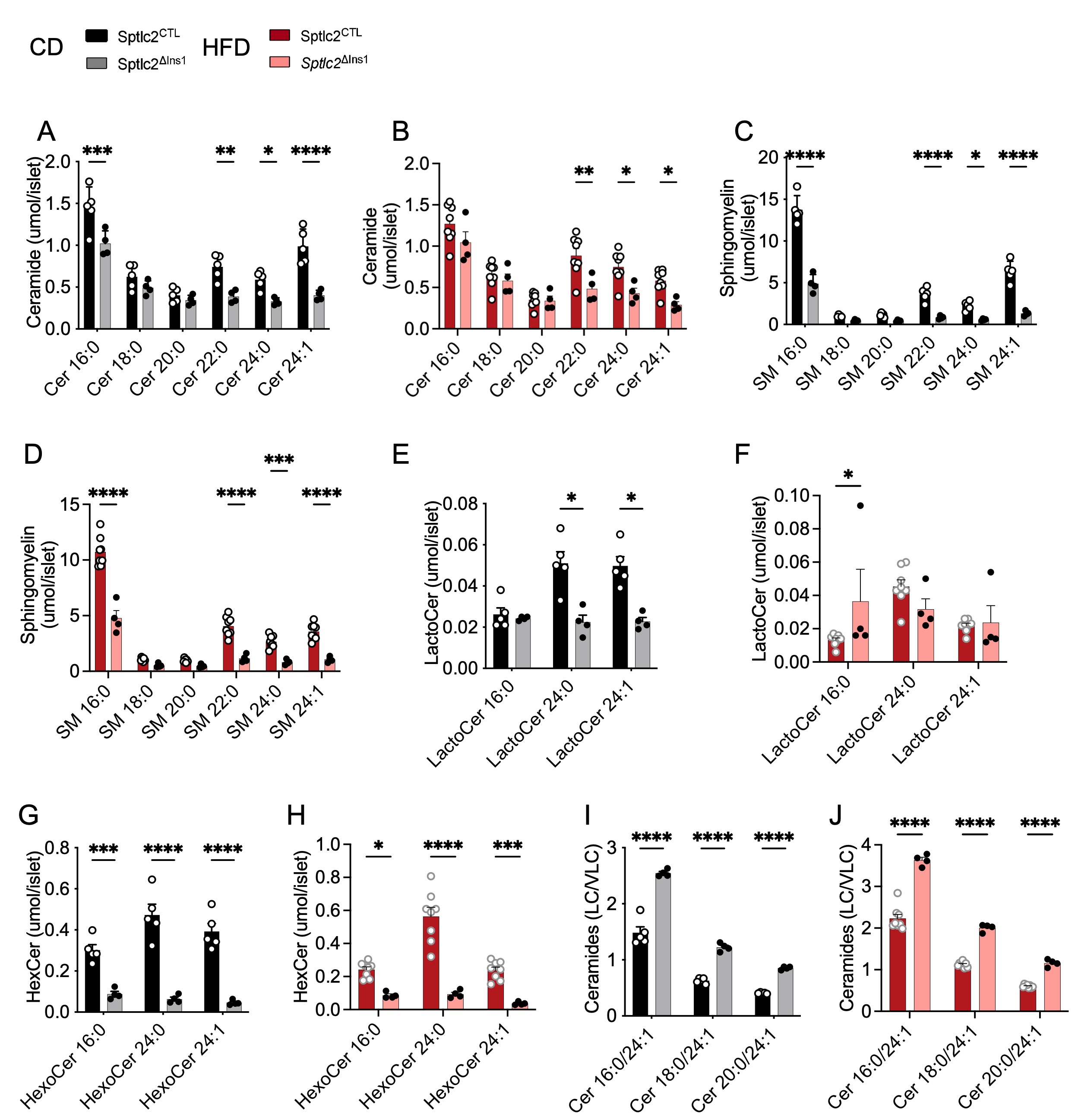

Supplement: Supplement 1 [file media-1.jpg]

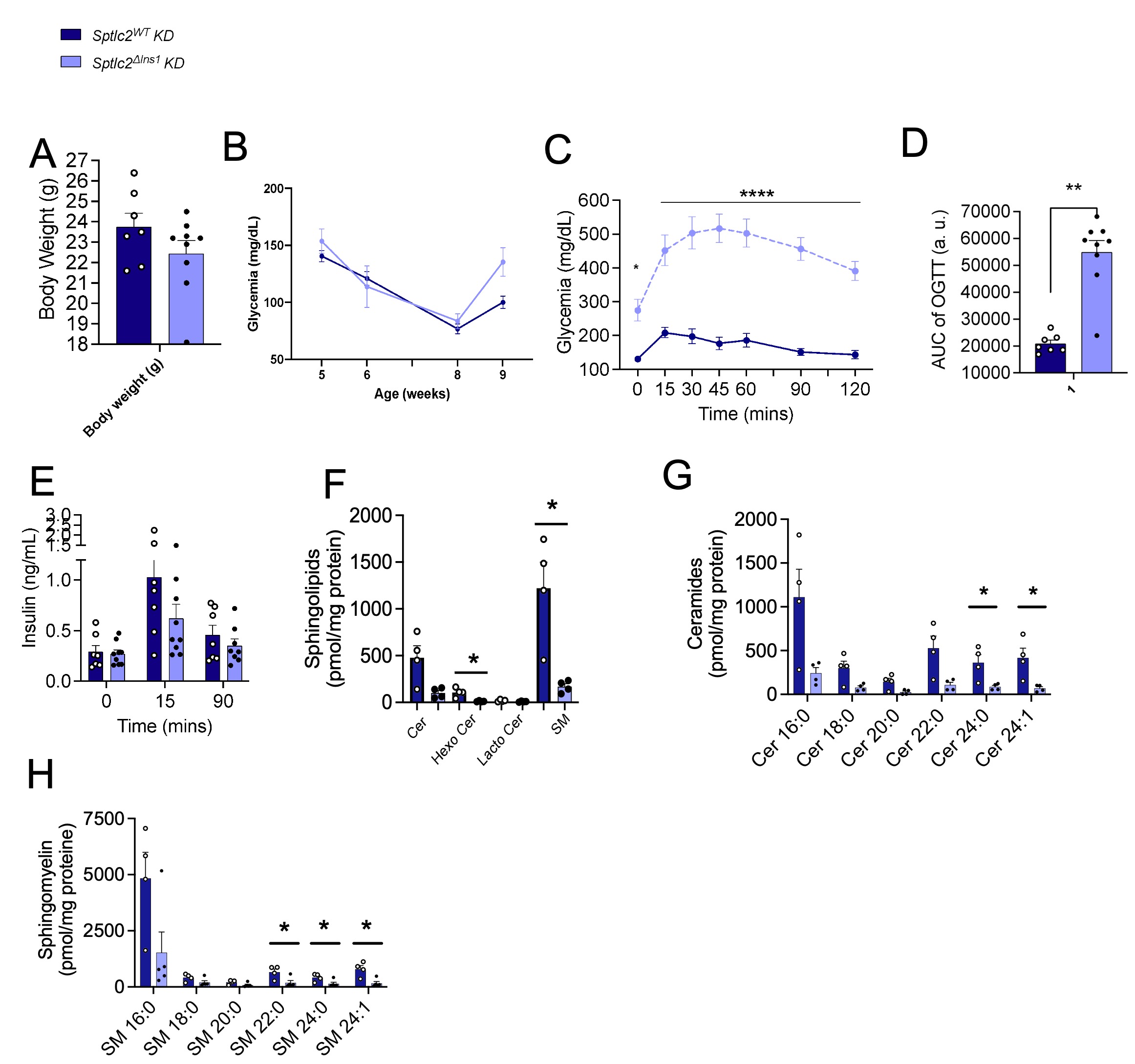

Supplement: Supplement 2 [file media-2.jpg]

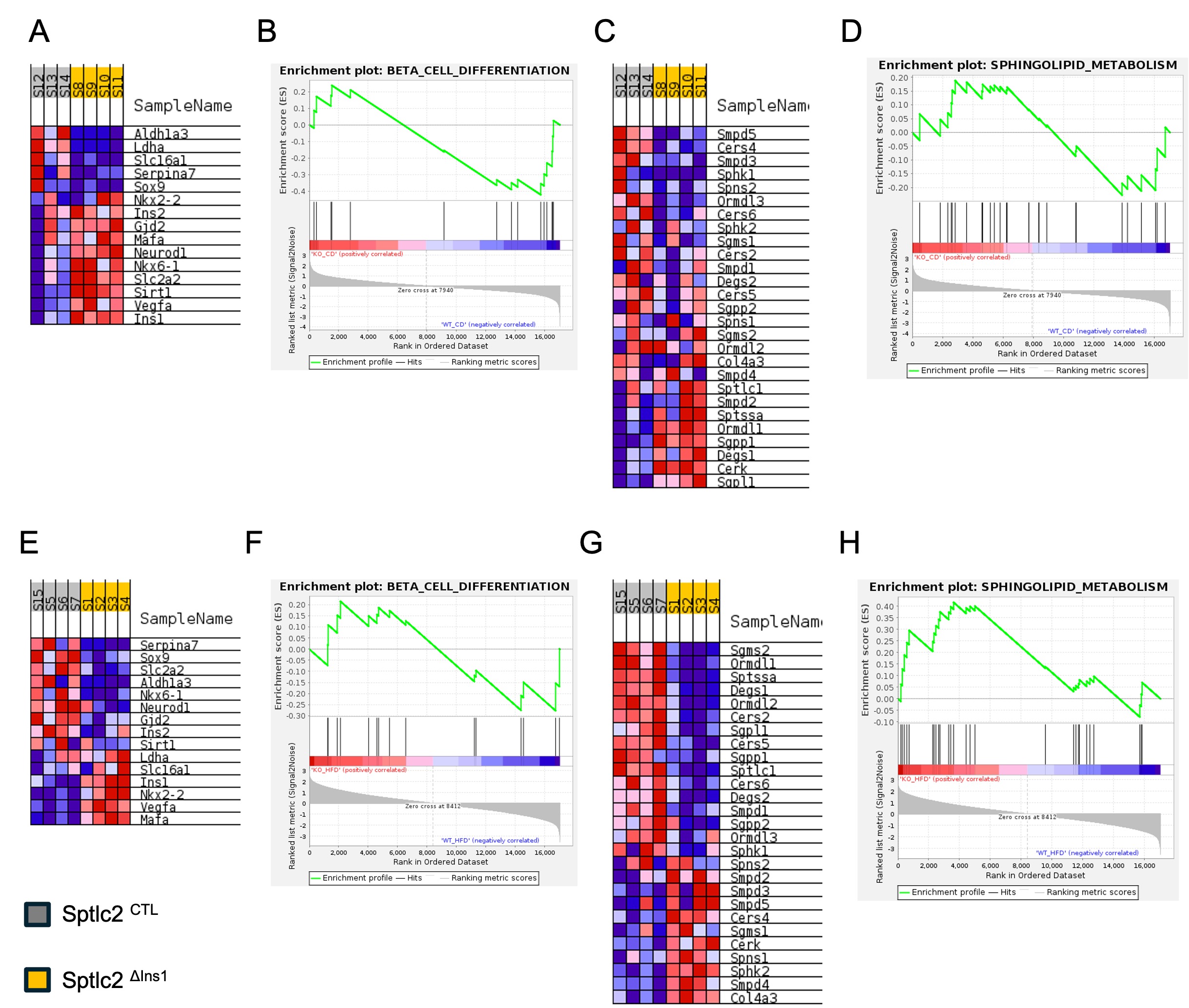

Supplement: Supplement 3 [file media-3.jpg]
